# Metagenomic survey of fungal communities in compost from dairy plant wastewater sludge and garden trimmings

**DOI:** 10.3897/BDJ.14.e174893

**Published:** 2026-04-09

**Authors:** Paulo Monjardino, Ana Rita Azevedo, Duarte Mendonça, Gabor Pozsgai, Paulo A. V. Borges, Jorge Frias, Duarte Toubarro

**Affiliations:** 1 University of the Azores, CBA – Centre of Biotecnology of the Azores, School of Agricultural and Environmental Sciences, Rua Capitão João d’Ávila, Pico da Urze, 9700-042, Angra do Heroísmo, Azores, Portugal University of the Azores, CBA – Centre of Biotecnology of the Azores, School of Agricultural and Environmental Sciences, Rua Capitão João d’Ávila, Pico da Urze, 9700-042 Angra do Heroísmo, Azores Portugal; 2 University of Azores, CE3C—Centre for Ecology, Evolution and Environmental Changes, Azorean Biodiversity Group, CHANGE —Global Change and Sustainability Institute, School of Agricultural and Environmental Sciences, Rua Capitão João d’Ávila, Pico da Urze, 9700-042, Angra do Heroísmo, Azores, Portugal University of Azores, CE3C—Centre for Ecology, Evolution and Environmental Changes, Azorean Biodiversity Group, CHANGE —Global Change and Sustainability Institute, School of Agricultural and Environmental Sciences, Rua Capitão João d’Ávila, Pico da Urze, 9700-042 Angra do Heroísmo, Azores Portugal; 3 IUCN SSC Monitoring Specialist Group, Angra do Heroísmo, Azores, Portugal IUCN SSC Monitoring Specialist Group Angra do Heroísmo, Azores Portugal; 4 University of the Azores, CBA – Centre of Biotecnology of the Azores, Rua Madre de Deus, 9500-321, Ponta Delgada, Azores, Portugal University of the Azores, CBA – Centre of Biotecnology of the Azores, Rua Madre de Deus, 9500-321 Ponta Delgada, Azores Portugal

**Keywords:** Thermophilic, cooling and maturation, garden trimmings, wastewater sludge, microbiome

## Abstract

**Background:**

Composting converts organic residues into stable organic matter and nutrients under aerobic conditions, improving soil properties and microbiome balance, while mitigating environmental impacts. Although microbiomes of various compost types have been studied, information is still fragmented and often not tailored to specific raw material combinations. In particular, little is known about the fungal communities involved in composting dairy plant wastewater sludge mixed with garden trimmings. This data paper contributes to filling that gap by providing a comprehensive taxonomic inventory.

**New information:**

We provide a fungus-focused dataset from 18 compost samples generated from a 1:1 (w/w) mix of garden trimmings and dairy plant wastewater sludge, collected at three process stages (thermophilic start/end; mid-cooling and maturation) under two turning regimes. Shotgun metagenomes were taxonomically annotated against NCBI taxonomy (accessed 19 Feb 2025). Only Fungi were detected within Eukarya, spanning nine phyla; Ascomycota (60.8%), Mucoromycota (17.76%), Basidiomycota (8.50%) and Chytridiomycota (7.21%) comprised 94.27% of the taxonomic features. We report 417 genera (13 >1% relative abundance each); top: *Aspergillus* (17.93%), *Rhizopus* (8.61%), *Chaetomium* (4.83%), *Aureobasidium* (3.09%), *Madurella* (2.85%), *Paramicrosporidium* (2.71%), *Rhizophagus* (1.88%), *Rasamsonia* (1.81%), *Hyaloraphidium* (1.39%), *Thermochaetoides* (1.31%), *Talaromyces* (1.19%), *Trichoderma* (1.15%), *Podospora* (1.06%) comprised 49.81% of the taxonomic feature abundance. Overall 663 taxa were identified (578 species, 416 genera, 230 families, 106 orders, 48 classes and 9 phyla). The dataset (DwCA; 663 occurrences) is intended to serve as a reference for compost mycobiomes and will be available via GBIF (DOI 10.15468/nmpzwr).

## Introduction

Composting is an aerobic process, in which microorganisms decompose organic matter into more stable compounds. The resulting product can be applied in agriculture as an organic fertiliser that improves soil physical properties, enriches the microbiome and enhances crop health while also contributing to nutrient recycling. In addition, composting reduces the environmental impact of organic residue accumulation and lowers sanitary risks through the pasteurisation effect of the thermophilic stage, which eliminates most coliform bacteria (Lin et al. 2018).

Sewage sludge, when co-composted with lignocellulosic materials, produces a stabilied, nutrient-enriched product. Nitrogen-rich wastes, such as food residues, manure and sewage sludge (C/N = 10–20), are typically combined with carbon-rich biomass (C/N > 50), which balances the C/N ratio and improves aeration while reducing leachate (Lin et al. 2018, Tarpani et al. 2020, Hoang et al. 2022). Composting also facilitates the dissipation of organic contaminants, including polycyclic aromatic hydrocarbons, pharmaceuticals, antibiotic-resistance genes and microplastics (Hoang et al. 2022).

Compared with anaerobic digestion, lime treatment, pyrolysis or incineration, composting offers several advantages, including nutrient recovery, low investment and operational costs, suitability for small- and medium-scale facilities and the potential to substitute chemical fertilisers. However, disadvantages include variable greenhouse gas emissions, high energy demand, requirement for large processing areas, limited volume reduction and no energy recovery (Hoang et al. 2022, Yu et al. 2023).

Microbial activity during composting is stage-dependent: bacteria dominate during the mesophilic and thermophilic phases, whereas fungi become more active during cooling and maturation (Tuomela et al. 2000). Labile compounds, such as sugars, starch, amino acids and lipids, are degraded early, releasing CO₂ and ammonia, whereas more recalcitrant polymers (cellulose, hemicellulose and lignin) are broken down later, contributing to humus formation. Fungi play a particularly important role due to their extracellular enzyme production, which enables the degradation of complex polymers and facilitates microbial interactions within compost piles (Awasthi et al. 2023).

## General description

### Purpose

This dataset provides information on the fungal community associated with the composting process. Composting is a biologically driven degradation process in which organic matter is transformed into a stable and nutrient-rich product. Fungi play an essential role in this process because of their ability to degrade complex polymers such as cellulose, hemicellulose and lignin, thereby accelerating organic matter decomposition and influencing the final quality of compost. The dataset was generated to document the diversity and taxonomic composition of fungi present in the composting material. The present Data Paper does not aim to evaluate treatment and compost stage effects (which will be addressed in a future article), but rather to provide baseline information for applied microbiology, waste management and soil fertility studies.

## Project description

### Title

Metagenomic Survey of Compost Fungi from Terceira (Azores Archipelago)

### Personnel

The project was conceived by Paulo Monjardino.

Fieldwork: Cátia Pereira, Paulo Monjardino.

Database management: Paulo Monjardino.

Darwin Core Database management: Paulo A. V. Borges & Paulo Monjardino.

### Funding

VERCOCHAR - Vermicompost, compost y biochar, herramientas para la adaptación al cambio climático, la prevención y mitigación de los efectos derivados de los riesgos naturales en el medio agrícola y forestal, MAC2/3.5b/307, FCT—Fundação para a Ciência e a Tecnologia, I.P., project UIDB/05292/2025 DOI https://doi.org/10.54499/UID/05292/2025 and from the Azorean Regional Directorate of Science, Innovation and Development, through the PROSCIENTIA Incentive System, Ref. M1.1. A/FUNC.UI&D/016/2025).

## Sampling methods

### Sampling description

**Compost Production**

Composting was performed at an open-air, industrial-scale facility operated by Teramb – Empresa Municipal de Gestão e Valorização Ambiental da Ilha Terceira, EM (38.67583° N, −27.17433° W). Wastewater sludge from a dairy processing plant was mixed with garden trimmings at a 1:1 (w/w) ratio. Garden trimmings were made of trees and shrubs branches (no more than 15 cm diameter), palm leaves, grass cuttings and garden herbaceous ornamentals. Two windrow-turning regimes were applied: (i) triweekly during the thermophilic stage and weekly during the cooling and maturation stages and (ii) once every three weeks throughout all stages.

**Sample Collection and DNA Extraction**

Samples were collected at the beginning and end of the thermophilic stage and at the mid-cooling and maturation. Material was obtained by digging 50–60 cm into the piles, ≈ 1 m above the ground, from three zones (external and middle) and bulked into composite samples. Each sample (200–300 g) was placed in a sterile bag and transported to the laboratory.

Samples were homogenied in PBS (pH 7.4), centrifuged, and the pellets were stored at −80 °C. Metagenomic DNA was extracted using the repeated bead-beating method (Yu and Morrison 2018) combined with a Quick-DNA Miniprep Plus Kit (Zymo Research 2017). DNA concentration and purity were measured using a NanoDrop spectrophotometer (Thermo Scientific, USA) and stored at −20 °C.

**Shotgun Sequencing**

Shotgun metagenomic sequencing was performed by Novogene (Cambridge, UK) on the Illumina NovaSeq 6000 platform (Illumina, San Diego, CA, USA), which provides high accuracy, scalability, and sequencing depth for complex microbiome studies (NOVOGENE 2023).

Genomic DNA (200 ng) was randomly sheared into ≈ 350 bp fragments using a Covaris ultrasonic disruptor. Libraries were prepared using Novogene NGS DNA Library Prep Set (Cat. No. PT004). Briefly, the fragmented DNA underwent end repair and A-tailing to produce blunt ends with adenine overhangs, followed by ligation of Illumina sequencing adapters containing unique barcodes. Size selection was performed to retain fragments of 350–450 bp by using AMPure XP beads (Beckman Coulter, USA). To enrich adapter-ligated fragments, PCR amplification was carried out when DNA input was low; for high-input samples, PCR-free preparation was applied to minimise bias.

Library quality was verified by fragment integrity and insert size analysis (AATI) and the effective library concentration was quantified by qPCR (> 3 nM). Qualified libraries were pooled in equimolar amounts and sequenced using the Illumina NovaSeq 6000 platform with two-channel sequencing-by-synthesis (SBS) chemistry. Paired-end sequencing (PE150) was employed to provide high-accuracy reads and improve the assembly performance, with error rates below 1%.

**Bioinformatics Analysis**

Raw reads were filtered using Trimmomatic v.0.39 to obtain high-quality clean data for downstream analysis. Reads from each sample were assembled individually to recover information from low-abundance species and the unused reads were pooled for mixed assembly using the metaSPAdes v.4.1.0 assembler. Gene prediction was carried out using MetaGeneMark v.3.25, based on the scaftigs that were assembled by single and mixed samples. The predicted genes were pooled and dereplicated to construct a non-redundant gene catalogue. Gene abundance for each sample was quantified by mapping reads back to this catalogue using a read mapper (Bowtie v.2.5.4). Taxonomic annotation was obtained for metagenomic reads against the microNR database, a curated collection of taxonomically informative gene families, using sequence alignment software called DIAMOND v.2.1.12. Taxon-specific abundance profiles were inferred from gene abundance estimates and aggregated to generate taxonomic feature tables at multiple taxonomic ranks. The taxonomic hierarchy of all fungal taxa was standardised and updated according to the NCBI Taxonomy database (accessed April 2025) using the taxize v.0.10.0 package in R v.4.5.1.

The data presented in this article originate from a composite analysis of 18 samples and represent the mean value across the entire sample set.

## Geographic coverage

### Description

Terceira Island, Azores Archipelago, Portugal. Solid waste processing centre managed by Teramb – Empresa Municipal de Gestão e Valorização Ambiental da Ilha Terceira, EM. In Terceira Island (Azores Archipelago).

### Coordinates

38.676 and 38.676 Latitude; -27.174 and -27.174 Longitude.

## Taxonomic coverage

### Description

The following Phyla are covered: Ascomycota, Basidiomycota, Blastocladiomycota, Chytridiomycota, Cryptomycota, Microsporidia, Mucoromycota, Olpidiomycota, Zoopagomycota.

The following Classes are covered: Agaricomycetes, Arthoniomycetes, Basidiobolomycetes, Blastocladiomycetes, Candelariomycetes, Chytridiomycetes, Coniocybomycetes, Dacrymycetes, Dimargaritomycetes, Dipodascomycetes, Dothideomycetes, Endogonomycetes, Entomophthoromycetes, Eurotiomycetes, Exobasidiomycetes, Geoglossomycetes, Glomeromycetes, Harpellomycetes, Kickxellomycetes, Lecanoromycetes, Leotiomycetes, Lichinomycetes, Lipomycetes, Malasseziomycetes, Microbotryomycetes, Mixiomycetes, Monoblepharidomycetes, Mortierellomycetes, Mucoromycetes, Neolectomycetes, Neocallimastigomycetes, Olpidiomycetes, Orbiliomycetes, Pezizomycetes, Physodermatomycetes, Pichiomycetes, Pneumocystomycetes, Pucciniomycetes, Saccharomycetes, Schizosaccharomycetes, Sordariomycetes, Taphrinomycetes, Tremellomycetes, Umbelopsidomycetes, Ustilaginomycetes, Wallemiomycetes, Xylobotryomycetes, Zoopagomycetes.

### Taxa included

**Table:** 

Rank	Scientific Name	Common Name
kingdom	Fungi	Fungi

## Temporal coverage

**Data range:** 2022-3-14 – 2022-11-28.

### Notes

The presented data were obtained from 18 samples collected between March 14, 2022 and November 28, 2022. DNA extraction was performed between March 8 and May 19, 2023, and sequencing analysis was completed in August 2023 (exact date unknown).

## Usage licence

### Usage licence

Creative Commons Public Domain Waiver (CC-Zero)

## Data resources

### Data package title

Metagenomic Survey of Compost Fungi from Terceira (Azores Archipelago)

### Resource link

https://ipt.gbif.pt/ipt/resource?r=compost_eukarya

### Alternative identifiers

https://www.gbif.org/dataset/e3b9f473-b41e-427e-89fc-990c973c8e05

### Number of data sets

1

### Data set 1.

#### Data set name

Occurrence Table

#### Data format

Darwin Core Archive

#### Character set

UTF-8

#### Download URL

https://ipt.gbif.pt/ipt/resource?r=compost_eukarya

#### Data format version

1.3

#### Description

The dataset was published in the Global Biodiversity Information Facility platform, GBIF (Monjardino et al. 2026). The following data-table includes all the records for which a taxonomic identification of the species was possible. The dataset submitted to GBIF is structured as a sample occurrence dataset that has been published as a Darwin Core Archive (DwCA), which is a standardised format for sharing biodiversity data as a set of one or more data tables. The core data file contains 663 records (eventID; and occurrenceID). This GBIF IPT (Integrated Publishing Toolkit, Version 2.5.6) archives the data and, thus, serves as the data repository. The data and resource metadata are available for download in the Portuguese GBIF Portal IPT (Monjardino et al. 2026).

**Data set 1.** 

Column label	Column description
id	Identifier generated by GBIF IPT.
type	The nature or genre of the resource.
licence	Reference to the licence under which the record is published.
institutionID	An identifier for the institution publishing the data.
institutionCode	The code of the institution publishing the data.
basisOfRecord	The nature of the data record (MaterialSample).
occurrenceID	Identifier of the record, coded as a global unique identifier.
recordedBy	A list (concatenated and separated) of names of people, groups or organisations who performed the sampling in the field.
organismQuantity	A number or enumeration value for the quantity of Organisms.
organismQuantityType	The type of quantification system used for the quantity of organisms.
eventID	Identifier of the events, unique for the dataset
eventDate	The date-time or interval during which an Event occurred.
year	Year the sample was collected.
eventRemarks	Comments or notes about the dwc:Event.
habitat	The surveyed habitat, in this case, a waste management site.
samplingProtocol	The sampling protocol used to capture the species.
sampleSizeValue	A numeric value for a measurement of the size (time duration, length, area or volume) of a sample in a sampling dwc:Event.
sampleSizeUnit	The unit of measurement of the size (time duration, length, area or volume) of a sample in a sampling dwc:Event.
locationID	Identifier of the location.
islandGroup	The name of the island group in which the Location occurs (Azores Archipelago).
island	The name of the island on which the Location occurs (Terceira).
country	The full, unabbreviated name of the next smaller administrative region than stateProvince (county, shire, department etc.) in which the Location occurs (Portugal).
countryCode	The standard code for the country in which the Location occurs (PT).
stateProvince	The name of the next smaller administrative region than country (state, province, canton, department, region etc.) in which the Location occurs.
municipality	The full, unabbreviated name of the next smaller administrative region than county (city, municipality etc.) in which the Location occurs.
locality	The specific description of the place.
minimumElevationInMetres	The lower limit of the range of elevation (altitude, usually above sea level), in metres.
decimalLatitude	Approximate centre point decimal latitude of the field site in GPS coordinates.
decimalLongitude	Approximate centre point decimal longitude of the field site in GPS coordinates.
geodeticDatum	Standard Global Positioning System coordinate reference for the location of the sample collection points.
coordinateUncertaintyInMetres	Uncertain value of coordinate metrics.
coordinatePrecision	Value in decimal degrees to a precision of six decimal places.
georeferenceSources	Navigation system used to record the location of sample collections.
identifiedBy	A list of names of people, groups or organisations who assigned the Taxon to the subject.
dateIdentified	The date on which the subject was determined as representing the Taxon.
identificationRemarks	Comments or notes about the dwc:Identification.
scientificName	The full scientific name, with authorship and date information if known.
kingdom	Kingdom name.
phylum	Phylum name.
class	Class name.
order	Order name.
family	Family name.
genus	Genus name.
specificEpithet	Specific epithet name.
taxonRank	Lowest taxonomic rank of the record.
scientificNameAuthorship	The authorship information for the scientificName formatted according to the conventions of the applicable nomenclaturalCode.

## Additional information

The microbiome analysis revealed four major groups: Eukarya, Archaea, Bacteria and Viruses (NCBI 2025). This study focuses exclusively on the Eukarya domain.

Within Eukarya, only the Fungi kingdom was detected, representing 0.01–0.1% of all taxonomic features and ranked as the least abundant group in 16 of the 18 compost samples. No other eukaryotic organisms (e.g. invertebrates, protozoa and algae) were detected. The absence of non-fungal eukaryotes in our dataset is likely related to strong ecological filtering during the thermophilic phase of composting. Temperatures during this stage commonly exceed 55–65°C, creating prolonged heat stress conditions that eliminate or severely reduce most non-spore-forming eukaryotes (Lin et al. 2018). Many fungal taxa are capable of surviving such conditions through thermotolerance mechanisms, rapid stress-response systems or the production of resistant structures such as spores (Tuomela et al. 2000). The thermophilic phase also accelerates organic matter degradation and alters oxygen availability and moisture dynamics, further restricting the establishment of other eukaryotic groups. Temperature-driven ecological filtering is a key determinant of compost microbial succession (Awasthi et al. 2023). The eukaryotic community may already have undergone strong selective filtering, resulting in the dominance of fungi. Aditionally, the very low overall proportion of Eukarya-derived reads (0.01–0.1%) suggests that other eukaryotic groups, if present, may fall below detection thresholds in shotgun metagenomic datasets dominated by bacterial and archaeal DNA (Quince et al. 2017).

Previous studies have similarly reported a relatively low abundance of fungi during composting (Martins et al. 2013, Wang et al. 2022, Aguilar-Paredes et al. 2023), suggesting that, while fungi contribute to the composting process, bacteria consistently dominate the microbial community. Although fungi represent a relatively small fraction of total metagenomic reads, their ecological importance in composting is disproportionate to their numerical abundance. Fungi are key degraders of recalcitrant polymers, such as cellulose, hemicellulose and lignin during both the thermophilic and subsequent cooling and maturation stages (Mello et al. 2017, Varma et al. 2017).

The present dataset provides an extensive inventory of fungal taxa identified in compost. While many have previously been reported in composting systems, others were not found in Web of Science, Scopus, Google Scholar or GBIF searches. This may reflect database limitations, as taxa not explicitly cited in titles, abstracts or indexed keywords are easily overlooked. Records from Google Scholar and GBIF can complement this gap, but require validation due to lower precision.

Another important consideration is the evolving state of fungal taxonomy. Over the past decade, major revisions have occurred following the abandonment of dual nomenclature and the adoption of molecular methods, which have clarified earlier misclassifications (Kidd et al. 2023). Consequently, even recent articles (as late as 2024) may report taxa using outdated classification. Taxonomic databases, including the NCBI database (NCBI 2025), are frequently updated. Between our first survey in 2023 and the present, 40–50 taxa have undergone reclassification, mainly at the species, genus and family levels and, in some cases, at the order and class levels.

The dominance of Ascomycota (Table 1) is consistent with other composting studies (Lin et al. 2018, Awasthi et al. 2023). However, the relatively high abundance of Mucoromycota, Basidiomycota and Chytridiomycota, which ranked second, third and fourth in taxonomic feature abundance, respectively, has not been reported elsewhere. Factors, such as raw material composition, mixture ratio, aeration, C/N balance, moisture, pH, shredding of garden trimmings and local environmental conditions, strongly affect the compost microbiome and may explain these differences. In metabolic terms, these fungal phyla play distinct roles during composting. Ascomycota and Mucoromycota contribute to the rapid degradation of simple carbohydrates and hemicellulose, Basidiomycota are particularly important lignin degraders through oxidative enzymes such as laccases and peroxidases and Chytridiomycota participate in the hydrolysis of cellulose and other recalcitrant polymers (Tuomela et al. 2000, Awasthi et al. 2023). Nevertheless, all phyla identified in this study have been reported previously in compost (Tuomela et al. 2000, Cai et al. 2018, Lin et al. 2018, He et al. 2022, Awasthi et al. 2023, Liu et al. 2024, Mahongnao et al. 2024, Ahmad et al. 2024).

At the genus level, the most abundant taxa (relative abundance of classified reads > 1%) were *Aspergillus*, *Rhizopus*, *Chaetomium*, *Aureobasidium*, *Madurella*, *Paramicrosporidium*, *Rhizophagus*, *Rasamsonia*, *Hyaloraphidium*, *Thermochaetoides*, *Talaromyces*, *Trichoderma* and *Podospora* (Table 2). These genera can be broadly grouped according to their metabolic or ecological roles during composting: (i) decomposers and nutrient cyclers (*Aspergillus*, *Rhizopus*, *Podospora*, *Thermochaetoides*); (ii) biocontrol and cellulolytic fungi (*Trichoderma*, *Chaetomium*, *Talaromyces*, *Rasamsonia*); (iii) yeasts and opportunistic fungi (*Aureobasidium*, *Madurella*); (iv) microsporidia and chytrids associated with aquatic or anaerobic niches (*Paramicrosporidium, Hyaloraphidium*) and (v) mycorrhizal fungi (*Rhizophagus*). Most of these genera have been previously reported in compost or during composting (Tuomela et al. 2000, Sebők et al. 2015, Anastasi et al. 2017, Cai et al. 2018, Lu et al. 2024, Mahongnao et al. 2024, Ahmad et al. 2024). *Paramicrosporidium* and *Hyaloraphidium* have been reported in non-aerated compost tea (Mengesha et al. 2017) and *Paramicrosporidium* was also detected in the gut of *Hermetia illucens* larvae grown in urban compost (Vallejo-Arróliga and Rojas-Jimenez 2024), suggesting that both genera may be associated with the composting environment.

Amongst the 13 most abundant genera, *Rhizophagus* (an arbuscular mycorrhizal fungus - AMF) is the only one without published reports linking it directly to composting. However, its presence in soil is well documented and studies have shown that compost addition stimulates hyphal growth and sporulation, benefiting multiple crops (Yang et al. 2018, Boutasknit et al. 2020, Tahiri et al. 2022). *Rhizophagus*, an obligate arbuscular mycorrhizal fungus (AMF), requires a living plant host for active growth (Smith and Read 2008). These fungi produce resistant spores capable of persisting in soil and plant residues and may be introduced into compost through yard trimmings or soil particles (Aguilar-Paredes et al. 2023). Shotgun metagenomic sequencing detects environmental DNA (eDNA) irrespective of metabolic state. Nevertheless, the observed relative abundance of 1.88% (within Fungi) suggests that additional factors may be involved beyond the mechanisms currently identified. The potential survival of AMF propagules during composting may have implications for the agricultural use of the final product.

Amongst the detected taxa (Monjardino et al. 2025), some correspond to host-specific or host-obligate parasites, including human-associated *Pneumocystis jirovecii* (Cissé et al. 2020), insect-associated *Entomophthora muscae* and *Massospora cicadina* (Elya and De Fine Licht 2021), amphibian-associated *Batrachochytrium salamandrivorans* (Martel et al. 2013) and amoeba-associated *Paramicrosporidium saccamoebae* (Quandt et al. 2017). Shotgun metagenomics identifies DNA fragments based on sequence similarity and does not discriminate amongst active organisms, dormant propagules or residual environmental DNA. Their detection may, therefore, reflect resistant spores, residual host material introduced through garden trimmings, undetected microscopic hosts or taxonomic assignments to closely-related environmental lineages. However, for relatively abundant taxa, such as *Batrachochytrium salamandrivorans* (RA = 0.59%) and *Paramicrosporidium saccamoebae* (RA = 2.71%) and, given that their known host species were not identified, additional factors may account for their presence in the composting material. These findings highlight the complexity of compost-associated microbial diversity and warrant further ecological investigation.

Although composting typically requires no inoculation, several studies demonstrated positive effects of fungal amendments, despite fungi often being amongst the least abundant microbial groups (He et al. 2022). Different taxa have been inoculated for: (i) detoxification of inhibitory compounds, for example, supplementation with *Paecilomyces* sp. FA13 enhances the degradation of furan derivatives during food waste pretreatment (Nakasaki et al. 2015); (ii) lignocellulose degradation and humification, with *Phanerochaete chrysosporium* and *Trichoderma longibrachiatum* increasing cellulose, hemicellulose and lignin breakdown, while elevating humus and humic acid content, all of which were also detected in our samples (Table 2); and (iii) synergistic effects with other microbes, such as inoculation with thermotolerant actinomycetes, which accelerates humification and increases humic substance content by 50–100% (Zhao et al. 2017).

Most of the fungi listed in Table 2 are relevant to composting. Based on previous studies and their relative abundances (> 0.5% of classified reads), three main functional groups emerge with the greatest potential for agricultural applications: (i) biocontrol fungi (*Trichoderma, Chaetomium, Aureobasidium, Penicillium, Paecilomyces*); (ii) mycorrhizal fungi (*Rhizophagus, Diversispora, Ambispora*) and (iii) nutrient cyclers and decomposers (*Aspergillus, Rhizopus, Mucor, Podospora, Thermothelomyces, Linnemannia*). The remaining genera detected were either pathogenic or of limited agricultural value.

In summary, this study provides a shotgun metagenomic inventory of fungal taxa detected across composting stages, documenting their relative read abundance and reinforcing the ecological and agricultural relevance of fungal communities within compost systems.

## Figures and Tables

**Table 1.** List of phyla detected during the composting process, with corresponding classified reads aggregated at phylum level and relative abundances (RA) within the Eukarya domain, confirmed in the NCBI Taxonomy Browser.

Phylum	Classified reads	RA
Ascomycota	135741	60.80%
Basidiomycota	18971	8.50%
Blastocladiomycota	1068	0.48%
Chytridiomycota	16096	7.21%
Cryptomycota	6508	2.92%
Microsporidia	2318	1.04%
Mucoromycota	39638	17.76%
Olpidiomycota	98	0.04%
Zoopagomycota	2802	1.26%
Total	223241	

**Table 2.** List of classes, orders, families and genera detected during the composting process, with corresponding classified reads counts and relative abundances (RA) within the Eukarya domain, confirmed in the NCBI Taxonomy Browser.

class	order	family	genus	Classified reads	RA
Dothideomycetes	Pleosporales		* Aaosphaeria *	48	0.02%
Mucoromycetes	Mucorales	Cunninghamellaceae	* Absidia *	269	0.12%
Exobasidiomycetes	Exobasidiales	Cryptobasidiaceae	* Acaromyces *	17	0.01%
Glomeromycetes	Diversisporales	Acaulosporaceae	* Acaulospora *	96	0.04%
Dothideomycetes	Mycosphaerellales	Teratosphaeriaceae	* Acidomyces *	23	0.01%
Mortierellomycetes	Mortierellales	Mortierellaceae	* Actinomortierella *	91	0.04%
Agaricomycetes	Agaricales	Agaricaceae	* Agaricus *	34	0.02%
Agaricomycetes	Agaricales	Strophariaceae	* Agrocybe *	48	0.02%
Dothideomycetes	Pleosporales	Pleosporaceae	* Alternaria *	60	0.03%
Arthoniomycetes	Arthoniales	Lecanographaceae	* Alyxoria *	29	0.01%
Glomeromycetes	Archaeosporales	Ambisporaceae	* Ambispora *	1196	0.54%
Dothideomycetes	Pleosporales	Amniculicolaceae	* Amniculicola *	59	0.03%
		Amphiacanthidae	* Amphiamblys *	17	0.01%
Leotiomycetes	Helotiales		* Amylocarpus *	38	0.02%
Neocallimastigomycetes	Neocallimastigales	Neocallimastigaceae	* Anaeromyces *	104	0.05%
Sordariomycetes	Xylariales	Hypoxylaceae	* Annulohypoxylon *	1963	0.88%
Dothideomycetes	Botryosphaeriales	Aplosporellaceae	* Aplosporella *	62	0.03%
Mucoromycetes	Mucorales	Mucoraceae	* Apophysomyces *	1266	0.57%
Orbiliomycetes	Orbiliales	Orbiliaceae	* Arthrobotrys *	389	0.17%
Eurotiomycetes	Onygenales	Arthrodermataceae	* Arthroderma *	18	0.01%
Agaricomycetes	Agaricales	Lyophyllaceae	* Arthromyces *	18	0.01%
Eurotiomycetes	Onygenales	Ascosphaeraceae	* Ascosphaera *	31	0.01%
Eurotiomycetes	Eurotiales	Aspergillaceae	* Aspergillus *	40031	17.93%
Agaricomycetes	Agaricales	Lyophyllaceae	* Asterophora *	26	0.01%
Agaricomycetes	Boletales	Astraeaceae	* Astraeus *	787	0.35%
Agaricomycetes	Atheliales	Atheliaceae	* Athelia *	62	0.03%
Dothideomycetes	Dothideales	Saccotheciaceae	* Aureobasidium *	6896	3.09%
Lecanoromycetes	Lecanorales	Ramalinaceae	* Bacidia *	19	0.01%
Mucoromycetes	Mucorales	Backusellaceae	* Backusella *	30	0.01%
Basidiobolomycetes	Basidiobolales	Basidiobolaceae	* Basidiobolus *	220	0.10%
Chytridiomycetes	Rhizophydiales		* Batrachochytrium *	2052	0.92%
Sordariomycetes	Hypocreales	Cordycipitaceae	* Beauveria *	627	0.28%
Mucoromycetes	Mucorales	Mucoraceae	* Benjaminiella *	20	0.01%
Endogonomycetes	Endogonales		* Bifiguratus *	328	0.15%
Blastocladiomycetes	Blastocladiales	Blastocladiaceae	* Blastocladiella *	490	0.22%
Eurotiomycetes	Onygenales	Ajellomycetaceae	* Blastomyces *	276	0.12%
Chytridiomycetes			* Blyttiomyces *	132	0.06%
Agaricomycetes	Boletales	Boletaceae	* Boletus *	39	0.02%
Agaricomycetes	Russulales	Bondarzewiaceae	* Bondarzewia *	171	0.08%
Chytridiomycetes	Rhizophydiales	Terramycetaceae	* Boothiomyces *	53	0.02%
Chytridiomycetes	Rhizophlyctidales	Borealophlyctidaceae	* Borealophlyctis *	131	0.06%
Leotiomycetes	Helotiales	Sclerotiniaceae	* Botryotinia *	22	0.01%
Leotiomycetes	Helotiales	Sclerotiniaceae	* Botrytis *	230	0.10%
Pichiomycetes	Pichiales	Pichiaceae	* Brettanomyces *	25	0.01%
Leotiomycetes	Helotiales	Ploettnerulaceae	* Cadophora *	51	0.02%
Dacrymycetes	Dacrymycetales	Dacrymycetaceae	* Calocera *	415	0.19%
Sordariomycetes	Hypocreales	Nectriaceae	* Calonectria *	203	0.09%
Candelariomycetes	Candelariales	Candelariaceae	* Candelaria *	194	0.09%
Pichiomycetes	Serinales	Debaryomycetaceae	* Candida *	794	0.36%
Agaricomycetes	Cantharellales	Hydnaceae	* Cantharellus *	42	0.02%
Entomophthoromycetes	Entomophthorales	Ancylistaceae	* Capillidium *	110	0.05%
Blastocladiomycetes	Blastocladiales	Catenariaceae	* Catenaria *	89	0.04%
Sordariomycetes	Microascales	Microascaceae	* Cephalotrichum *	876	0.39%
Agaricomycetes	Cantharellales	Ceratobasidiaceae	* Ceratobasidium *	997	0.45%
Sordariomycetes	Microascales	Ceratocystidaceae	* Ceratocystis *	127	0.06%
Glomeromycetes	Diversisporales	Gigasporaceae	* Cetraspora *	863	0.39%
Sordariomycetes	Sordariales	Chaetomiaceae	* Chaetomium *	10780	4.83%
Mucoromycetes	Mucorales	Choanephoraceae	* Choanephora *	71	0.03%
Agaricomycetes	Boletales	Gomphidiaceae	* Chroogomphus *	50	0.02%
Sordariomycetes	Diaporthales	Cryphonectriaceae	* Chrysoporthe *	1935	0.87%
Chytridiomycetes	Chytridiales	Chytridiaceae	* Chytridium *	21	0.01%
Chytridiomycetes	Chytridiales	Chytriomycetaceae	* Chytriomyces *	1061	0.48%
Mucoromycetes	Mucorales	Lichtheimiaceae	* Circinella *	409	0.18%
Xylobotryomycetes	Xylobotryales	Cirrosporiaceae	* Cirrosporium *	48	0.02%
Sordariomycetes	Hypocreales	Hypocreaceae	* Cladobotryum *	184	0.08%
Chytridiomycetes	Cladochytriales	Cladochytriaceae	* Cladochytrium *	408	0.18%
Eurotiomycetes	Chaetothyriales	Herpotrichiellaceae	* Cladophialophora *	103	0.05%
Sordariomycetes	Hypocreales	Clavicipitaceae	* Claviceps *	43	0.02%
Pichiomycetes	Serinales	Metschnikowiaceae	* Clavispora *	18	0.01%
Dothideomycetes	Pleosporales	Lindgomycetaceae	* Clohesyomyces *	48	0.02%
Chytridiomycetes	Lobulomycetales	Lobulomycetaceae	* Clydaea *	20	0.01%
Eurotiomycetes	Onygenales	Onygenaceae	* Coccidioides *	200	0.09%
Blastocladiomycetes	Blastocladiales	Coelomomycetaceae	* Coelomomyces *	137	0.06%
Kickxellomycetes	Kickxellales	Kickxellaceae	* Coemansia *	322	0.14%
Leotiomycetes	Helotiales	Dermateaceae	* Coleophoma *	109	0.05%
Sordariomycetes	Sordariales	Chaetomiaceae	* Collariella *	114	0.05%
Sordariomycetes	Glomerellales	Glomerellaceae	* Colletotrichum *	1965	0.88%
Entomophthoromycetes	Entomophthorales	Ancylistaceae	* Conidiobolus *	107	0.05%
Sordariomycetes	Coniochaetales	Coniochaetaceae	* Coniochaeta *	169	0.08%
Dothideomycetes	Pleosporales	Coniothyriaceae	* Coniothyrium *	623	0.28%
Agaricomycetes	Agaricales	Psathyrellaceae	* Coprinellus *	56	0.03%
Dothideomycetes	Pleosporales	Corynesporascaceae	* Corynespora *	88	0.04%
Agaricomycetes	Agaricales	Crepidotaceae	* Crepidotus *	18	0.01%
Sordariomycetes	Diaporthales	Cryphonectriaceae	* Cryphonectria *	196	0.09%
Tremellomycetes	Tremellales	Cryptococcaceae	* Cryptococcus *	112	0.05%
			* Cucumispora *	341	0.15%
Leotiomycetes	Helotiales	Tricladiaceae	* Cudoniella *	6	0.00%
Mucoromycetes	Mucorales	Cunninghamellaceae	* Cunninghamella *	24	0.01%
Agaricomycetes	Agaricales	Nidulariaceae	* Cyathus *	64	0.03%
Dacrymycetes	Dacrymycetales	Dacrymycetaceae	* Dacryopinax *	58	0.03%
Orbiliomycetes	Orbiliales	Orbiliaceae	* Dactylella *	518	0.23%
Sordariomycetes	Hypocreales	Nectriaceae	* Dactylonectria *	23	0.01%
Sordariomycetes	Xylariales	Hypoxylaceae	* Daldinia *	630	0.28%
Pichiomycetes	Serinales	Debaryomycetaceae	* Debaryomyces *	303	0.14%
Agaricomycetes	Agaricales		* Dendrothele *	49	0.02%
Glomeromycetes	Diversisporales	Gigasporaceae	* Dentiscutata *	85	0.04%
Sordariomycetes	Diaporthales	Diaporthaceae	* Diaporthe *	78	0.03%
Agaricomycetes	Polyporales	Polyporaceae	* Dichomitus *	77	0.03%
Mucoromycetes	Mucorales	Lichtheimiaceae	* Dichotomocladium *	146	0.07%
			* Dictyocoela *	69	0.03%
Dimargaritomycetes	Dimargaritales	Dimargaritaceae	* Dimargaris *	169	0.08%
Chytridiomycetes	Chytridiales	Chytridiaceae	* Dinochytrium *	76	0.03%
Leotiomycetes	Helotiales	Drepanopezizaceae	* Diplocarpon *	37	0.02%
Lecanoromycetes	Ostropales	Graphidaceae	* Diploschistes *	128	0.06%
Kickxellomycetes	Kickxellales	Kickxellaceae	* Dipsacomyces *	120	0.05%
Mortierellomycetes	Mortierellales	Mortierellaceae	* Dissophora *	62	0.03%
Glomeromycetes	Diversisporales	Diversisporaceae	* Diversispora *	1695	0.76%
Orbiliomycetes	Orbiliales	Orbiliaceae	* Drechslerella *	108	0.05%
Sordariomycetes	Xylariales	Xylariaceae	* Durotheca *	28	0.01%
Eurotiomycetes	Eurotiales	Elaphomycetaceae	* Elaphomyces *	304	0.14%
Eurotiomycetes	Onygenales	Ajellomycetaceae	* Emergomyces *	17	0.01%
Sordariomycetes	Hypocreales	Bionectriaceae	* Emericellopsis *	144	0.06%
Endogonomycetes	Endogonales	Endogonaceae	* Endogone *	418	0.19%
			* Enteropsectra *	55	0.02%
		Enterocytozoonidae	* Enterospora *	84	0.04%
Entomophthoromycetes	Entomophthorales	Entomophthoraceae	* Entomophthora *	30	0.01%
Mortierellomycetes	Mortierellales	Mortierellaceae	* Entomortierella *	67	0.03%
Chytridiomycetes	Chytridiales	Chytriomycetaceae	* Entophlyctis *	23	0.01%
Glomeromycetes	Entrophosporales	Entrophosporaceae	* Entrophospora *	322	0.14%
Dothideomycetes	Eremomycetales	Eremomycetaceae	* Eremomyces *	152	0.07%
Saccharomycetes	Saccharomycetales	Saccharomycetaceae	* Eremothecium *	13	0.01%
Leotiomycetes	Erysiphales	Erysiphaceae	* Erysiphe *	509	0.23%
Agaricomycetes	Auriculariales	Exidiaceae	* Exidia *	47	0.02%
Exobasidiomycetes	Exobasidiales	Exobasidiaceae	* Exobasidium *	15	0.01%
Eurotiomycetes	Chaetothyriales	Herpotrichiellaceae	* Exophiala *	61	0.03%
Dothideomycetes	Pleosporales	Pleosporaceae	* Exserohilum *	873	0.39%
Tremellomycetes	Filobasidiales	Filobasidiaceae	* Filobasidium *	256	0.11%
Chytridiomycetes	Spizellomycetales	Powellomycetaceae	* Fimicolochytrium *	42	0.02%
Agaricomycetes	Agaricales	Tricholomataceae	* Flagelloscypha *	18	0.01%
Agaricomycetes	Hymenochaetales	Hymenochaetaceae	* Fomitiporia *	314	0.14%
Agaricomycetes	Polyporales	Fomitopsidaceae	* Fomitopsis *	62	0.03%
Eurotiomycetes	Chaetothyriales	Herpotrichiellaceae	* Fonsecaea *	65	0.03%
Dothideomycetes	Mycosphaerellales	Teratosphaeriaceae	* Friedmanniomyces *	595	0.27%
Glomeromycetes	Glomerales	Glomeraceae	* Funneliformis *	1048	0.47%
Sordariomycetes	Hypocreales	Nectriaceae	* Fusarium *	599	0.27%
Chytridiomycetes	Spizellomycetales	Spizellomycetaceae	* Gaertneriomyces *	90	0.04%
Mortierellomycetes	Mortierellales	Mortierellaceae	* Gamsiella *	63	0.03%
Agaricomycetes	Polyporales	Polyporaceae	* Ganoderma *	37	0.02%
Sordariomycetes	Sordariales	Sordariaceae	* Gelasinospora *	68	0.03%
Geoglossomycetes	Geoglossales	Geoglossaceae	* Geoglossum *	469	0.21%
Glomeromycetes	Archaeosporales	Geosiphonaceae	* Geosiphon *	80	0.04%
Dipodascomycetes	Dipodascales	Dipodascaceae	* Geotrichum *	373	0.17%
Chytridiomycetes	Spizellomycetales	Powellomycetaceae	* Geranomyces *	126	0.06%
Glomeromycetes	Diversisporales	Gigasporaceae	* Gigaspora *	1068	0.48%
Chytridiomycetes	Rhizophydiales	Globomycetaceae	* Globomyces *	276	0.12%
Glomeromycetes	Glomerales	Glomeraceae	* Glomus *	40	0.02%
Geoglossomycetes	Geoglossales	Geoglossaceae	* Glutinoglossum *	359	0.16%
Sordariomycetes	Diaporthales	Gnomoniaceae	* Gnomoniopsis *	19	0.01%
Leotiomycetes	Erysiphales	Erysiphaceae	* Golovinomyces *	297	0.13%
Lecanoromycetes	Ostropales	Graphidaceae	* Gomphillus *	297	0.13%
Monoblepharidomycetes	Monoblepharidales	Gonapodyaceae	* Gonapodya *	597	0.27%
Dothideomycetes	Acrospermales	Acrospermaceae	* Gonatophragmium *	87	0.04%
Mucoromycetes	Mucorales	Cunninghamellaceae	* Gongronella *	197	0.09%
Chytridiomycetes	Rhizophydiales	Gorgonomycetaceae	* Gorgonomyces *	1147	0.51%
Mortierellomycetes	Mortierellales	Mortierellaceae	* Gryganskiella *	40	0.02%
Lecanoromycetes	Teloschistales	Teloschistaceae	* Gyalolechia *	20	0.01%
Mucoromycetes	Mucorales	Cunninghamellaceae	* Halteromyces *	24	0.01%
Saccharomycetes	Saccharomycodales	Saccharomycodaceae	* Hanseniaspora *	44	0.02%
Mortierellomycetes	Mortierellales	Mortierellaceae	* Haplosporangium *	59	0.03%
Sordariomycetes	Hypocreales	Bionectriaceae	* Hapsidospora *	16	0.01%
Agaricomycetes	Agaricales	Hymenogastraceae	* Hebeloma *	330	0.15%
Eurotiomycetes	Onygenales	Ajellomycetaceae	* Helicocarpus *	111	0.05%
Agaricomycetes	Russulales	Hericiaceae	* Hericium *	218	0.10%
Agaricomycetes	Polyporales	Meruliaceae	* Hermanssonia *	76	0.03%
Agaricomycetes	Russulales	Bondarzewiaceae	* Heterobasidion *	35	0.02%
Sordariomycetes	Hypocreales	Ophiocordycipitaceae	* Hirsutella *	50	0.02%
Eurotiomycetes	Onygenales	Ajellomycetaceae	* Histoplasma *	1379	0.62%
Dothideomycetes	Mycosphaerellales	Teratosphaeriaceae	* Hortaea *	213	0.10%
Monoblepharidomycetes	Monoblepharidales		* Hyaloraphidium *	3105	1.39%
Leotiomycetes	Helotiales	Hyaloscyphaceae	* Hyaloscypha *	33	0.01%
Agaricomycetes	Boletales	Hygrophoropsidaceae	* Hygrophoropsis *	30	0.01%
Sordariomycetes	Xylariales	Hypoxylaceae	* Hypomontagnella *	12	0.01%
Sordariomycetes	Hypocreales	Hypocreaceae	* Hypomyces *	61	0.03%
Sordariomycetes	Xylariales	Hypoxylaceae	* Hypoxylon *	196	0.09%
Agaricomycetes	Agaricales	Lyophyllaceae	* Hypsizygus *	114	0.05%
Sordariomycetes	Hypocreales	Nectriaceae	* Ilyonectria *	270	0.12%
Agaricomycetes	Hymenochaetales	Hymenochaetaceae	* Inonotus *	39	0.02%
Chytridiomycetes	Chytridiales	Chytridiaceae	* Irineochytrium *	257	0.12%
Agaricomycetes	Polyporales	Irpicaceae	* Irpex *	89	0.04%
Agaricomycetes	Jaapiales	Jaapiaceae	* Jaapia *	65	0.03%
Endogonomycetes	Endogonales	Endogonaceae	* Jimgerdemannia *	61	0.03%
Sordariomycetes	Diaporthales	Juglanconidaceae	* Juglanconis *	1238	0.55%
Sordariomycetes	Microascales	Microascaceae	* Kernia *	297	0.13%
Tremellomycetes	Tremellales	Cryptococcaceae	* Kwoniella *	267	0.12%
Agaricomycetes	Agaricales	Hydnangiaceae	* Laccaria *	54	0.02%
Saccharomycetes	Saccharomycetales	Saccharomycetaceae	* Lachancea *	496	0.22%
Agaricomycetes	Polyporales	Laetiporaceae	* Laetiporus *	47	0.02%
Lecanoromycetes	Umbilicariales	Umbilicariaceae	* Lasallia *	24	0.01%
Agaricomycetes	Agaricales	Omphalotaceae	* Lentinula *	57	0.03%
Agaricomycetes	Polyporales	Polyporaceae	* Lentinus *	77	0.03%
Dothideomycetes	Pleosporales	Lentitheciaceae	* Lentithecium *	82	0.04%
Dothideomycetes	Mytilinidiales	Argynnaceae	* Lepidopterella *	70	0.03%
Leotiomycetes	Helotiales	Leptodontidiaceae	* Leptodontidium *	1666	0.75%
Sordariomycetes	Ophiostomatales	Ophiostomataceae	* Leptographium *	52	0.02%
Agaricomycetes	Boletales		* Leucogyrophana *	188	0.08%
Mucoromycetes	Mucorales	Lichtheimiaceae	* Lichtheimia *	561	0.25%
Kickxellomycetes	Kickxellales	Kickxellaceae	* Linderina *	42	0.02%
Mortierellomycetes	Mortierellales	Mortierellaceae	* Linnemannia *	1196	0.54%
Lipomycetes	Lipomycetales	Lipomycetaceae	* Lipomyces *	9	0.00%
Mortierellomycetes	Mortierellales	Mortierellaceae	* Lobosporangium *	21	0.01%
Chytridiomycetes	Lobulomycetales	Lobulomycetaceae	* Lobulomyces *	19	0.01%
Sordariomycetes	Microascales	Microascaceae	* Lomentospora *	38	0.02%
Dothideomycetes	Pleosporales	Lophiotremataceae	* Lophiotrema *	28	0.01%
Agaricomycetes	Agaricales	Lyophyllaceae	* Lyophyllum *	60	0.03%
Dothideomycetes	Botryosphaeriales	Botryosphaeriaceae	* Macrophomina *	1885	0.84%
Sordariomycetes	Sordariales		* Madurella *	6366	2.85%
Dipodascomycetes	Dipodascales	Dipodascaceae	* Magnusiomyces *	92	0.04%
Malasseziomycetes	Malasseziales	Malasseziaceae	* Malassezia *	236	0.11%
Entomophthoromycetes	Entomophthorales	Entomophthoraceae	* Massospora *	25	0.01%
Exobasidiomycetes	Exobasidiales	Brachybasidiaceae	* Meira *	434	0.19%
Sordariomycetes	Hypocreales	Clavicipitaceae	* Metarhizium *	340	0.15%
Pichiomycetes	Serinales	Metschnikowiaceae	* Metschnikowia *	40	0.02%
Entomophthoromycetes	Entomophthorales	Ancylistaceae	* Microconidiobolus *	198	0.09%
Sordariomycetes	Xylariales	Microdochiaceae	* Microdochium *	31	0.01%
			* Mitosporidium *	933	0.42%
Mixiomycetes	Mixiales	Mixiaceae	* Mixia *	11	0.00%
Sordariomycetes	Hypocreales	Clavicipitaceae	* Moelleriella *	104	0.05%
Leotiomycetes	Helotiales	Mollisiaceae	* Mollisia *	19	0.01%
Eurotiomycetes	Eurotiales	Aspergillaceae	* Monascus *	147	0.07%
Leotiomycetes	Helotiales	Sclerotiniaceae	* Monilinia *	771	0.35%
Agaricomycetes	Agaricales	Marasmiaceae	* Moniliophthora *	71	0.03%
Monoblepharidomycetes	Monoblepharidales	Monoblepharidaceae	* Monoblepharella *	512	0.23%
Sordariomycetes	Xylariales		* Monosporascus *	144	0.06%
Pezizomycetes	Pezizales	Morchellaceae	* Morchella *	1020	0.46%
Mortierellomycetes	Mortierellales	Mortierellaceae	* Mortierella *	855	0.38%
Mucoromycetes	Mucorales	Mucoraceae	* Mucor *	1315	0.59%
Agaricomycetes	Agaricales	Mycenaceae	* Mycena *	797	0.36%
Sordariomycetes	Sordariales	Chaetomiaceae	* Mycothermus *	12	0.01%
Mucoromycetes	Mucorales	Mycotyphaceae	* Mycotypha *	63	0.03%
Tremellomycetes	Filobasidiales	Filobasidiaceae	* Naganishia *	22	0.01%
Saccharomycetes	Saccharomycetales	Saccharomycetaceae	* Nakaseomyces *	59	0.03%
Eurotiomycetes	Onygenales	Arthrodermataceae	* Nannizzia *	30	0.01%
Sordariomycetes	Xylariales	Xylariaceae	* Nemania *	71	0.03%
			* Nematocida *	225	0.10%
Sordariomycetes	Xylariales	Apiosporaceae	* Neoarthrinium *	100	0.04%
Neocallimastigomycetes	Neocallimastigales	Neocallimastigaceae	* Neocallimastix *	50	0.02%
Entomophthoromycetes	Entomophthorales	Ancylistaceae	* Neoconidiobolus *	14	0.01%
Dothideomycetes	Mycosphaerellales	Teratosphaeriaceae	* Neohortaea *	48	0.02%
Neolectomycetes	Neolectales	Neolectaceae	* Neolecta *	41	0.02%
Sordariomycetes	Xylariales	Sporocadaceae	* Neopestalotiopsis *	106	0.05%
Sordariomycetes	Sordariales	Sordariaceae	* Neurospora *	619	0.28%
		Nosematidae	* Nosema *	382	0.17%
Chytridiomycetes	Cladochytriales	Nowakowskiellaceae	* Nowakowskiella *	175	0.08%
Chytridiomycetes	Chytridiales	Chytriomycetaceae	* Obelidium *	20	0.01%
Pichiomycetes	Pichiales	Pichiaceae	* Ogataea *	13	0.01%
Olpidiomycetes	Olpidiales	Olpidiaceae	* Olpidium *	98	0.04%
Sordariomycetes	Hypocreales	Ophiocordycipitaceae	* Ophiocordyceps *	858	0.38%
Sordariomycetes	Diaporthales	Gnomoniaceae	* Ophiognomonia *	456	0.20%
Sordariomycetes	Ophiostomatales	Ophiostomataceae	* Ophiostoma *	799	0.36%
Orbiliomycetes	Orbiliales	Orbiliaceae	* Orbilia *	1955	0.88%
Eurotiomycetes	Eurotiales	Thermoascaceae	* Paecilomyces *	1119	0.50%
Agaricomycetes	Agaricales	Galeropsidaceae	* Panaeolus *	197	0.09%
			* Pancytospora *	92	0.04%
Agaricomycetes	Polyporales	Panaceae	* Panus *	17	0.01%
Eurotiomycetes	Onygenales	Ajellomycetaceae	* Paracoccidioides *	392	0.18%
Glomeromycetes	Paraglomerales	Paraglomeraceae	* Paraglomus *	360	0.16%
Sordariomycetes	Hypocreales	Ophiocordycipitaceae	* Paraisaria *	40	0.02%
			* Paramicrosporidium *	6046	2.71%
Sordariomycetes	Hypocreales	Stachybotryaceae	* Paramyrothecium *	16	0.01%
Physodermatomycetes	Physodermatales	Physodermataceae	* Paraphysoderma *	353	0.16%
Mucoromycetes	Mucorales	Mucoraceae	* Parasitella *	602	0.27%
Agaricomycetes	Boletales	Paxillaceae	* Paxillus *	56	0.02%
Lecanoromycetes	Peltigerales	Peltigeraceae	* Peltigera *	188	0.08%
Lichinomycetes	Lichinales	Phylliscaceae	* Peltula *	78	0.04%
Eurotiomycetes	Eurotiales	Aspergillaceae	* Penicilliopsis *	539	0.24%
Eurotiomycetes	Eurotiales	Aspergillaceae	* Penicillium *	1845	0.83%
Agaricomycetes	Russulales	Peniophoraceae	* Peniophora *	312	0.14%
Sordariomycetes	Xylariales	Sporocadaceae	* Pestalotiopsis *	191	0.09%
Tremellomycetes	Cystofilobasidiales	Mrakiaceae	* Phaffia *	16	0.01%
Agaricomycetes	Polyporales	Phanerochaetaceae	* Phanerochaete *	207	0.09%
Mucoromycetes	Mucorales	Lichtheimiaceae	* Phascolomyces *	14	0.01%
Eurotiomycetes	Chaetothyriales	Herpotrichiellaceae	* Phialophora *	19	0.01%
Chytridiomycetes	Chytridiales	Chytridiaceae	* Phlyctochytrium *	822	0.37%
Agaricomycetes	Agaricales	Strophariaceae	* Pholiota *	53	0.02%
Mucoromycetes	Mucorales	Phycomycetaceae	* Phycomyces *	96	0.04%
Lichinomycetes	Lichinales	Phylliscaceae	* Phylliscum *	38	0.02%
Lecanoromycetes	Caliciales	Physciaceae	* Physcia *	30	0.01%
Agaricomycetes	Polyporales	Meripilaceae	* Physisporinus *	243	0.11%
Pichiomycetes	Pichiales	Pichiaceae	* Pichia *	111	0.05%
Mucoromycetes	Mucorales	Mucoraceae	* Pilaira *	29	0.01%
Mucoromycetes	Mucorales	Pilobolaceae	* Pilobolus *	61	0.03%
Zoopagomycetes	Zoopagales	Piptocephalidaceae	* Piptocephalis *	65	0.03%
Neocallimastigomycetes	Neocallimastigales	Neocallimastigaceae	* Piromyces *	619	0.28%
Sordariomycetes	Glomerellales	Plectosphaerellaceae	* Plectosphaerella *	1257	0.56%
Dothideomycetes	Pleosporales	Leptosphaeriaceae	* Plenodomus *	25	0.01%
Agaricomycetes	Agaricales	Pleurotaceae	* Pleurotus *	18	0.01%
Pneumocystomycetes	Pneumocystales	Pneumocystaceae	* Pneumocystis *	72	0.03%
Sordariomycetes	Hypocreales	Clavicipitaceae	* Pochonia *	106	0.05%
Mortierellomycetes	Mortierellales	Mortierellaceae	* Podila *	73	0.03%
Chytridiomycetes	Chytridiales	Chytriomycetaceae	* Podochytrium *	12	0.01%
Leotiomycetes	Erysiphales	Erysiphaceae	* Podosphaera *	161	0.07%
Sordariomycetes	Sordariales	Podosporaceae	* Podospora *	2356	1.06%
Chytridiomycetes	Polychytriales		* Polychytrium *	248	0.11%
Dothideomycetes	Pleosporales	Tetraplosphaeriaceae	* Polyplosphaeria *	47	0.02%
Chytridiomycetes	Rhizophydiales		* Polyrhizophydium *	73	0.03%
Eurotiomycetes	Onygenales		* Polytolypa *	74	0.03%
Chytridiomycetes	Spizellomycetales	Powellomycetaceae	* Powellomyces *	264	0.12%
Dothideomycetes	Mycosphaerellales	Mycosphaerellaceae	* Pseudocercospora *	14	0.01%
Leotiomycetes	Thelebolales	Thelebolaceae	* Pseudogymnoascus *	819	0.37%
			* Pseudoloma *	48	0.02%
Ustilaginomycetes	Ustilaginales	Ustilaginaceae	* Pseudozyma *	24	0.01%
Agaricomycetes	Agaricales	Strophariaceae	* Psilocybe *	561	0.25%
Pucciniomycetes	Pucciniales	Pucciniaceae	* Puccinia *	483	0.22%
Pezizomycetes	Pezizales	Pyronemataceae	* Pyronema *	306	0.14%
Chytridiomycetes		Quaeritorhizaceae	* Quaeritorhiza *	1731	0.78%
Glomeromycetes	Diversisporales	Gigasporaceae	* Racocetra *	299	0.13%
Mucoromycetes	Mucorales	Radiomycetaceae	* Radiomyces *	61	0.03%
Agaricomycetes	Gomphales	Gomphaceae	* Ramaria *	103	0.05%
	Ramicandelaberales	Ramicandelaberaceae	* Ramicandelaber *	34	0.02%
Dothideomycetes	Mycosphaerellales	Mycosphaerellaceae	* Ramularia *	62	0.03%
Eurotiomycetes	Eurotiales	Trichocomaceae	* Rasamsonia *	4036	1.81%
Chytridiomycetes	Chytridiales	Chytriomycetaceae	* Rhizoclosmatium *	962	0.43%
Agaricomycetes	Cantharellales	Ceratobasidiaceae	* Rhizoctonia *	758	0.34%
Dothideomycetes	Aulographales	Rhizodiscinaceae	* Rhizodiscina *	15	0.01%
Glomeromycetes	Glomerales	Glomeraceae	* Rhizophagus *	4204	1.88%
Chytridiomycetes	Rhizophlyctidales	Rhizophlyctidaceae	* Rhizophlyctis *	462	0.21%
Agaricomycetes	Boletales	Rhizopogonaceae	* Rhizopogon *	47	0.02%
Mucoromycetes	Mucorales	Rhizopodaceae	* Rhizopus *	19211	8.61%
Agaricomycetes	Agaricales	Omphalotaceae	* Rhodocollybia *	33	0.01%
Agaricomycetes	Polyporales	Fomitopsidaceae	* Rhodofomes *	117	0.05%
Agaricomycetes	Polyporales	Adustoporiaceae	* Rhodonia *	493	0.22%
Microbotryomycetes	Sporidiobolales	Sporidiobolaceae	* Rhodotorula *	107	0.05%
Sordariomycetes	Xylariales	Xylariaceae	* Rosellinia *	154	0.07%
			* Rozella *	462	0.21%
Agaricomycetes	Russulales	Russulaceae	* Russula *	942	0.42%
Leotiomycetes	Helotiales	Rutstroemiaceae	* Rutstroemia *	38	0.02%
Saccharomycetes	Saccharomycetales	Saccharomycetaceae	* Saccharomyces *	542	0.24%
Saccharomycetes	Saccharomycodales	Saccharomycodaceae	* Saccharomycodes *	31	0.01%
			* Saitoella *	284	0.13%
Tremellomycetes	Tremellales	Trimorphomycetaceae	* Saitozyma *	792	0.35%
Agaricomycetes	Hymenochaetales	Hymenochaetaceae	* Sanghuangporus *	202	0.09%
Agaricomycetes	Agaricales	Schizophyllaceae	* Schizophyllum *	100	0.04%
Agaricomycetes	Hymenochaetales	Schizoporaceae	* Schizopora *	42	0.02%
Schizosaccharomycetes	Schizosaccharomycetales	Schizosaccharomycetaceae	* Schizosaccharomyces *	22	0.01%
Agaricomycetes	Boletales	Sclerodermataceae	* Scleroderma *	28	0.01%
Coniocybomycetes	Coniocybales	Coniocybaceae	* Sclerophora *	198	0.09%
Leotiomycetes	Helotiales	Sclerotiniaceae	* Sclerotinia *	281	0.13%
Sordariomycetes	Microascales	Microascaceae	* Scopulariopsis *	1907	0.85%
Glomeromycetes	Diversisporales	Gigasporaceae	* Scutellospora *	21	0.01%
Leotiomycetes			* Scytalidium *	740	0.33%
Agaricomycetes	Sebacinales	Serendipitaceae	* Serendipita *	345	0.15%
Agaricomycetes	Boletales	Serpulaceae	* Serpula *	23	0.01%
Harpellomycetes	Harpellales	Legeriomycetaceae	* Smittium *	423	0.19%
Sordariomycetes	Sordariales	Sordariaceae	* Sordaria *	752	0.34%
Agaricomycetes	Geastrales	Sphaerobolaceae	* Sphaerobolus *	88	0.04%
Pezizomycetes	Pezizales	Pyronemataceae	* Sphaerosporella *	13	0.01%
Kickxellomycetes	Kickxellales	Kickxellaceae	* Spiromyces *	77	0.03%
Chytridiomycetes	Spizellomycetales	Spizellomycetaceae	* Spizellomyces *	123	0.06%
Dothideomycetes	Pleosporales	Massarinaceae	* Stagonospora *	24	0.01%
Sordariomycetes	Sordariales	Chaetomiaceae	* Staphylotrichum *	350	0.16%
Agaricomycetes	Agaricales	Strophariaceae	* Stropharia *	181	0.08%
Agaricomycetes	Boletales	Suillaceae	* Suillus *	313	0.14%
Mucoromycetes	Mucorales	Syncephalastraceae	* Syncephalastrum *	51	0.02%
Zoopagomycetes	Zoopagales	Piptocephalidaceae	* Syncephalis *	781	0.35%
Chytridiomycetes	Synchytriales	Synchytriaceae	* Synchytrium *	187	0.08%
Eurotiomycetes	Eurotiales	Trichocomaceae	* Talaromyces *	2651	1.19%
Taphrinomycetes	Taphrinales	Taphrinaceae	* Taphrina *	30	0.01%
Dothideomycetes	Mycosphaerellales	Teratosphaeriaceae	* Teratosphaeria *	2041	0.91%
Pezizomycetes	Pezizales	Pezizaceae	* Terfezia *	47	0.02%
Agaricomycetes	Agaricales	Lyophyllaceae	* Termitomyces *	1006	0.45%
Chytridiomycetes	Rhizophydiales	Terramycetaceae	* Terramyces *	37	0.02%
Agaricomycetes	Agaricales	Marasmiaceae	* Tetrapyrgos *	183	0.08%
Mucoromycetes	Mucorales	Mucoraceae	* Thamnidium *	125	0.06%
Lecanoromycetes	Thelocarpales	Thelocarpaceae	* Thelocarpon *	22	0.01%
Sordariomycetes	Sordariales	Chaetomiaceae	* Thermochaetoides *	2930	1.31%
Eurotiomycetes	Eurotiales	Trichocomaceae	* Thermomyces *	172	0.08%
Sordariomycetes	Sordariales	Chaetomiaceae	* Thermothelomyces *	1758	0.79%
Sordariomycetes	Sordariales	Chaetomiaceae	* Thermothielavioides *	1116	0.50%
Sordariomycetes	Chaetosphaeriales	Chaetosphaeriaceae	* Thozetella *	28	0.01%
Sordariomycetes		Thyridiaceae	* Thyridium *	317	0.14%
Exobasidiomycetes	Tilletiales	Tilletiaceae	* Tilletia *	100	0.04%
Exobasidiomycetes	Entylomatales		* Tilletiopsis *	14	0.01%
Sordariomycetes	Hypocreales	Ophiocordycipitaceae	* Tolypocladium *	23	0.01%
Saccharomycetes	Saccharomycetales	Saccharomycetaceae	* Torulaspora *	69	0.03%
Dothideomycetes	Venturiales	Cylindrosympodiaceae	* Tothia *	477	0.21%
Agaricomycetes	Polyporales	Polyporaceae	* Trametes *	723	0.32%
Agaricomycetes	Polyporales	Irpicaceae	* Trametopsis *	16	0.01%
Tremellomycetes	Tremellales	Tremellaceae	* Tremella *	70	0.03%
Dothideomycetes	Phaeotrichales	Phaeotrichaceae	* Trichodelitschia *	28	0.01%
Sordariomycetes	Hypocreales	Hypocreaceae	* Trichoderma *	2559	1.15%
Geoglossomycetes	Geoglossales	Geoglossaceae	* Trichoglossum *	49	0.02%
Agaricomycetes	Agaricales	Lyophyllaceae	* Tricholomella *	37	0.02%
Eurotiomycetes	Onygenales	Arthrodermataceae	* Trichophyton *	149	0.07%
Tremellomycetes	Trichosporonales	Trichosporonaceae	* Trichosporon *	33	0.01%
Leotiomycetes	Helotiales	Helotiaceae	* Tricladium *	24	0.01%
Pezizomycetes	Pezizales	Tuberaceae	* Tuber *	764	0.34%
		Tubulinosematidae	* Tubulinosema *	15	0.01%
Agaricomycetes	Cantharellales	Tulasnellaceae	* Tulasnella *	303	0.14%
Agaricomycetes	Agaricales	Psathyrellaceae	* Tulosesus *	73	0.03%
Agaricomycetes	Boletales	Boletaceae	* Tylopilus *	446	0.20%
Umbelopsidomycetes	Umbelopsidales	Umbelopsidaceae	* Umbelopsis *	234	0.10%
Eurotiomycetes	Onygenales	Onygenaceae	* Uncinocarpus *	15	0.01%
Ustilaginomycetes	Ustilaginales	Ustilaginaceae	* Ustilago *	434	0.19%
Sordariomycetes	Xylariales	Xylariaceae	* Ustulina *	74	0.03%
		Nosematidae	* Vairimorpha *	27	0.01%
Tremellomycetes	Trichosporonales	Trichosporonaceae	* Vanrija *	53	0.02%
Agaricomycetes	Russulales	Lachnocladiaceae	* Vararia *	11	0.00%
Leotiomycetes	Helotiales	Discinellaceae	* Varicosporium *	31	0.01%
Dothideomycetes	Venturiales	Venturiaceae	* Venturia *	59	0.03%
Leotiomycetes	Helotiales	Pleuroascaceae	* Venustampulla *	126	0.06%
Dothideomycetes	Venturiales	Sympoventuriaceae	* Verruconis *	20	0.01%
Sordariomycetes	Glomerellales	Plectosphaerellaceae	* Verticillium *	231	0.10%
		Nosematidae	* Vittaforma *	30	0.01%
Agaricomycetes	Agaricales	Pluteaceae	* Volvariella *	17	0.01%
Wallemiomycetes	Wallemiales	Wallemiaceae	* Wallemia *	1304	0.58%
Lichinomycetes	Lichinales	Porocyphaceae	* Watsoniomyces *	33	0.01%
Dipodascomycetes	Dipodascales	Trichomonascaceae	* Wickerhamiella *	83	0.04%
Saccharomycetes	Phaffomycetales	Wickerhamomycetaceae	* Wickerhamomyces *	181	0.08%
Agaricomycetes	Polyporales	Phaeolaceae	* Wolfiporia *	44	0.02%
Sordariomycetes	Xylariales	Xylariaceae	* Xylaria *	591	0.26%
Sordariomycetes	Xylariales	Xylariaceae	* Xylariaceae *	31	0.01%
Leotiomycetes			* Xylogone *	108	0.05%
Pichiomycetes	Serinales	Debaryomycetaceae	* Yamadazyma *	22	0.01%
Harpellomycetes	Harpellales	Legeriomycetaceae	* Zancudomyces *	65	0.03%
Dothideomycetes		Zopfiaceae	* Zopfia *	117	0.05%
Mucoromycetes	Mucorales	Lichtheimiaceae	* Zychaea *	48	0.02%
Eurotiomycetes	Chaetothyriales			69	0.03%
Chytridiomycetes	Chytridiales			62	0.03%
